# Pattern Recognition Analysis of Proton Nuclear Magnetic Resonance Spectra of Brain Tissue Extracts from Rats Anesthetized with Propofol or Isoflurane

**DOI:** 10.1371/journal.pone.0011172

**Published:** 2010-06-17

**Authors:** Hiroshi Kawaguchi, Keiko Hirakawa, Kensuke Miyauchi, Kaoru Koike, Youkichi Ohno, Atsuhiro Sakamoto

**Affiliations:** 1 Department of Anesthesiology, Nippon Medical School, Bunkyo-ku, Tokyo, Japan; 2 NMR Laboratory and Department of Legal Medicine, Nippon Medical School, Bunkyo-ku, Tokyo, Japan; 3 Department of Primary Care and Emergency Medicine, Kyoto University Graduate School of Medicine, Kyoto, Japan; Queensland Brain Institute, Australia

## Abstract

**Background:**

General anesthesia is routinely used as a surgical procedure and its safety has been endorsed by clinical outcomes; however, its effects at the molecular level have not been elucidated. General anesthetics influence glucose metabolism in the brain. However, the effects of anesthetics on brain metabolites other than those related to glucose have not been well characterized. We used a pattern recognition analysis of proton nuclear magnetic resonance spectra to visualize the changes in holistic brain metabolic phenotypes in response to the widely used intravenous anesthetic propofol and the volatile anesthetic isoflurane.

**Methodology/Principal Findings:**

Rats were randomized into five groups (n = 7 each group). Propofol and isoflurane were administered to two groups each, for 2 or 6 h. The control group received no anesthesia. Brains were removed directly after anesthesia. Hydrophilic compounds were extracted from excised whole brains and measured by proton nuclear magnetic resonance spectroscopy. All spectral data were processed and analyzed by principal component analysis for comparison of the metabolite profiles. Data were visualized by plotting principal component (PC) scores. In the plots, each point represents an individual sample. The propofol and isoflurane groups were clustered separately on the plots, and this separation was especially pronounced when comparing the 6-h groups. The PC scores of the propofol group were clearly distinct from those of the control group, particularly in the 6-h group, whereas the difference in PC scores was more subtle in the isoflurane group and control groups.

**Conclusions/Significance:**

The results of the present study showed that propofol and isoflurane exerted differential effects on holistic brain metabolism under anesthesia.

## Introduction

General anesthetics act on the central nervous system, mainly the brain. Although general anesthesia has been routinely used as a surgical procedure and its safety has been evaluated and endorsed by clinical outcomes, its effects at the molecular level have not yet been clarified. Intravenous and inhalational anesthetics have differing effects on cerebral hemodynamics. Inhalational anesthetics cause some vasodilation, whereas intravenous anesthetics do not [1]–[5]. In addition, general anesthetics suppress the activity of brain glucose metabolism in animals and humans in a dose-dependent manner, and administration of a number of anesthetics suppress glucose utilization in the brain [6]–[8]. For example, as reported by Alkire et al., propofol and isoflurane both affect glucose metabolism of the human brain [9], [10]. Many other endogenous low molecular compounds are present in the brain tissue. However, the effect of administration of an anesthetic drug on their metabolic changes is not known in detail.

For elucidating cellular functions, an exhaustive analysis of the expression of genes (genomics) and proteins (proteomics) has been performed. Metabolomics or metabonomics, defined as the exhaustive analysis of endogenous metabolites, is an area of great interest for investigating the end products of gene expression. Metabonomics-based approaches are capable of measuring the dynamic multiparametric responses of living systems to internal and external influences [11].

Pattern recognition analysis of proton nuclear magnetic resonance (^1^H-NMR) spectra of various tissues is now widely used for metabolomic studies. This tool obtains metabolite profiles of tissues without the use of any process to separate the metabolites. A data set of the digitized spectra is analyzed by multivariate analysis, such as principal component analysis (PCA), and the pattern recognition contained within the spectra enables features of the metabolite profiles of the tissues to be extracted, even if all the metabolites detected are not assigned. Moreover, ^1^H-NMR spectroscopy is the only method that can measure many types of metabolites in a tissue or in an organism noninvasively and non-destructively.

We hypothesized that anesthetics change brain metabolism over time, and that this change is different depending on the type of compound. Our goal in this study was to compare the metabolic responses in the brain to anesthesia with representative intravenous (propofol) and volatile (isoflurane) anesthetics. We examined endogenous rat brain metabolite profiles using pattern recognition of ^1^H-NMR spectra with multivariate analysis, and visualized the holistic metabolic brain phenotype over time under anesthesia.

## Results

Following induction of propofol or isoflurane anesthesia, all the rats lost consciousness within 5 min, with loss of corneal reflexes, as well as withdrawal response to pain. Until the end of anesthesia, the rats from both groups continued to experience a loss in corneal reflexes and withdrawal response to pain.

**Figure 1:**
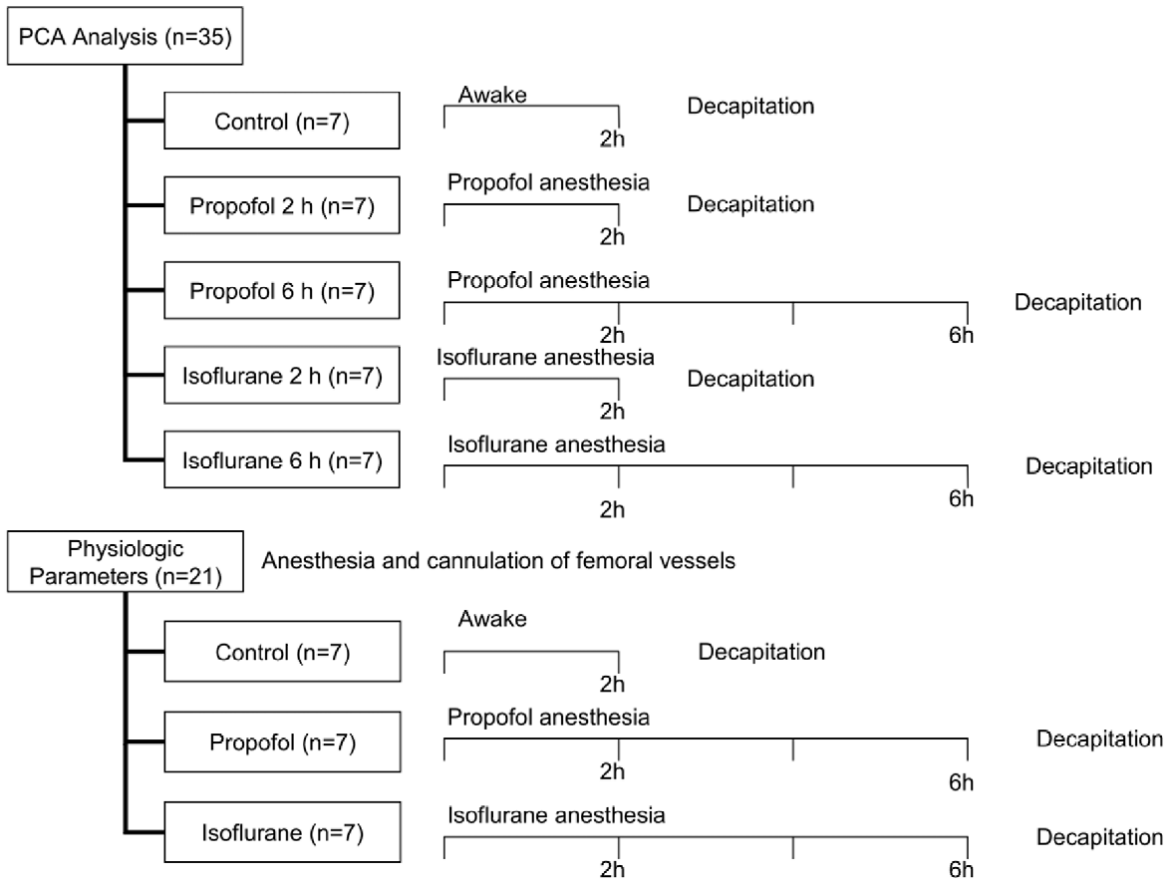
Flow chart of the experimental protocol.

We investigated the possible effects of anesthesia on the physiological parameters in three separate groups of rats. Figure 1 shows a flow chart of the experimental protocol. Physiological data during anesthesia were compared with those of the control group in which no anesthesia was administered after the termination of anesthesia for the cannulation of the femoral vessels. We observed low mean blood pressure and high PCO_2_ following the administration of anesthetic drugs. However, there were no significant differences in the administering group and the control group (Table 1).

**Table 1:** Physiological Parameters in Control and Anesthetized Rats.

	Control group	Propofol group		Isoflurane group	
		2h	6h	2h	6h
**pH**	7.45±0.01	7.43±0.04	7.42±0.02	7.43±0.03	7.41±0.03
**PaO2 (mmHg)**	106.7±7.5	109.8±15.8	101.9±18.3	103.1±11.6	111±7.6
**PCO2 (mmHg)**	37.4±1.5	43.1±3.5	43.5±3.6	41.5±3.2	44.7±1.6
**Heart rate (beats/min)**	304±29	323±24	291±30	293±31	294±21
**Mean arterial pressure (mmHg)**	109±5	98±18	94±8	100±6	95±10
**Plasma glucose concentration (mg/dl)**	140±16.4	152±23.7	162±18.0	143±10.5	148±12.9

Data represent mean ±SD for seven animals in each group.

**Figure 2:**
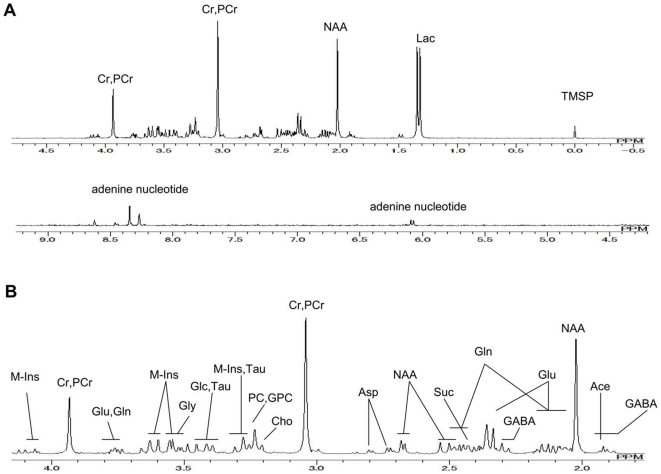
Proton NMR spectrum of a hydrophilic brain extract in a control rat. (A) 0.00 ppm to 9.00 ppm and (B) 2.00 ppm to 4.00 ppm (Ace. Acetate: Asp. Aspartate: Cho. Choline: Cr. Creatine: GABA. gamma-aminobutyric acid: Glc. Glucose: Gln. Glutamine: Glu. Glutamate: Gly. Glycine: GPC. Glycerophosphorylcholine: M-Ins. Myo Inositol: NAA. N-acetyl aspartate: PC. Phosphorylcholine: PCr. Phosphocreatine: Suc. Succinate: Tau. Taurine).

Figure 2 shows a typical proton NMR spectrum of hydrophilic extracts of the rat brain in the control group, with details regarding the main metabolites.

**Figure 3:**
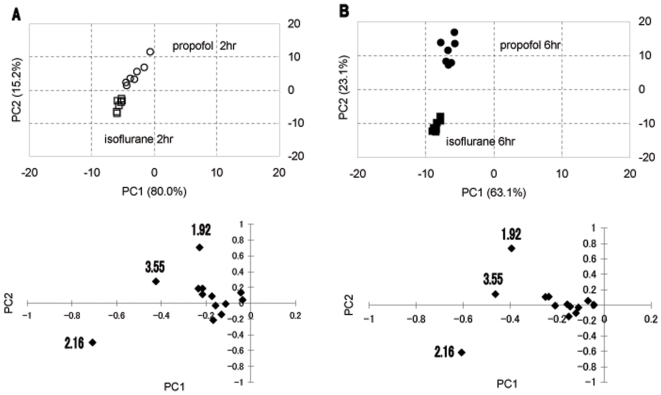
PCA score plot of hydrophilic brain extracts. Each mark represents the metabolic data set from an individual rat. (A) Propofol 2 h (○) and isoflurane 2 h (□). (B) Propofol 6 h (•) and isoflurane 6 h (▪). Variables (n = 15 reduced from 177). The lower graphs are PC loading plots of individual data sets to distinguish single putative NMR peaks responsible for the clustering pattern observed in the PCA score plot.

PCA is a multivariate technique that reduces highly dimensional data into only a few principal components (PCs). PCs are new variables created from linear combinations of the starting variables with appropriate weighting coefficients. The first PC (PC 1) contains the largest part of variance of the data set. In this manner, the data can be reduced into two dimensions, which allows graphic observation of trends in the data. Figure 3 shows the scores plot (PC 1 vs. 2) resulting from PCA. The plots for the propofol group and isoflurane group at 2 h and 6 h were clearly separately clustered, with a larger separation at 6 h (Figure 3B) than at 2 h (Figure 3A). Loading plots for PC1 vs. PC2 are also shown at the bottom of Figure 3. Loading plot shows the relative contribution to the PCs of the original variable. The numbers in the loading plots indicate the center value of the chemical shift of each of the buckets as follows: 1.92 ppm (acetate and GABA), 2.16 ppm (mainly glutamine and glutamate) and 3.55 ppm (glycine).

**Figure 4:**
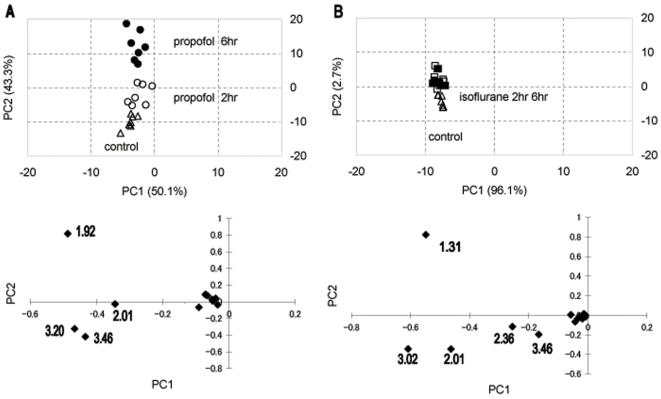
PCA score plot of hydrophilic brain extracts. Each mark represents the metabolic data set from an individual rat. (A) Control (▵), propofol 2 h (○), and propofol 6 h (•). (B) Control (▵), isoflurane 2 h (□) and isoflurane 6 h (▪). Variables (n = 1\educed from 177). The lower graphs are PC loading plots of individual data sets to distinguish single putative NMR peaks responsible for the clustering pattern observed in the PCA score plot.

The PCA scores plot revealed that the propofol 2 h, propofol 6 h, and control groups were clearly clustered with good separation among the groups (Figure 4A). Loading plots for PC1 vs. PC2 are also shown at the bottom of Figure 4. The numbers in the loading plots indicate the center value of the chemical shift of each of the buckets as follows: 1.92 ppm (mainly acetate), 2.01 ppm (N-acetyl aspartate (NAA)), 3.20 ppm (cholines) and 3.46 ppm (mainly glucose). The mean integral values of the buckets of 1.92 ppm, which included the acetate resonance, were significantly higher in the propofol groups than those in the control group (data not shown). The PCA scores plot showed that the isoflurane 2 h, isoflurane 6 h, and control groups were clustered among the groups (Figure 4B). The separation was clear between the isoflurane administered groups and the control group, but was subtle between the isoflurane 2 h group and isoflurane 6 h group compared with the propofol groups (Figure 4A). Loading plots for PC1 vs. PC2 are also shown at the bottom of Figure 4. The numbers in the loading plots indicate the center value of the chemical shift of each of the buckets as follows: 1.31 ppm (lactate), 2.01 ppm (N-acetyl aspartate (NAA)), 2.36 ppm (glutamate), 3.02 ppm (creatine) and 3.46 ppm (mainly glucose).

## Discussion

We found that brain metabolic phenotypes are different under different types of anesthesia, namely propofol or isoflurane. Furthermore, while we observed few changes in brain metabolism during the time course of anesthesia using isoflurane, there were remarkable changes during the time course of propofol anesthesia.

Prior to the development of various metabolomic approaches, NMR spectroscopy was often used for the analysis of metabolites from biological fluids and tissues. In particular, multivariate analysis (pattern recognition analysis) of proton NMR spectra can generate metabolite profiles of tissues [12], [13]. An NMR spectrum of a biological sample can be thought of as an object with a multidimensional set of metabolic coordinates, the values of which are the spectral intensities at each data point. Therefore, the spectrum is a point in multidimensional metabolic hyperspace. This allows the use of PCA [14] in metabolomics, which provides a snapshot of chronological changes in the biochemical state of tissues.

The NMR-based metabolomic approach that we used in our study is a chemometric technique that detects differences in the NMR spectrum pattern among samples from rat brain tissues by comparing the relative signal integrals within the same regions of chemical shifts in each spectrum. PCA was used to process the huge dataset obtained from this NMR approach. PCA was applied to map the metabolic phenotypes of samples from the control, propofol, and isoflurane groups, and to identify markers responsible for group clustering. The results revealed that propofol and isoflurane have different effects on holistic brain metabolism, and we successfully visualized these differences on PCA score plots.

Although the majority of NMR-based metabolomic studies are carried out using equipment with high magnetic field strength such as 600 MHz or above, the present study was carried out using a magnet with a field strength of 300 MHz (7 Tesla), which was capable of separating the metabolic phenotypes of the experimental groups with high sensitivity and specificity. Metabolic changes induced by anesthesia can be monitored using such equipment with low magnetic field strength. It is important to apply a proper statistical analysis post-data acquisition such as data reduction, feature extraction and visualization. Our experiments were aimed not at the accurate quantitation of individual NMR peaks, but rather a comparison of the patterns of spectra using chemometric methods that visualize the metabolic phenotypes of samples. Recent studies [15], [16] have shown good results with 300-MHz NMR equipment. *In vivo* NMR spectroscopy measurements on humans are widely performed using a 3T magnetic resonance imaging system.

In this study, we conducted experiments using propofol as an intravenous anesthetic and isoflurane as an inhaled anesthetic. Both these anesthetics are extensively used in everyday clinical practice. We evaluated the metabolomic changes in the brain of rats anesthetized with 1 minimum alveolar concentration of isoflurane or with continuous infusion of propofol at 40 mg/kg/h. These doses were chosen because lower doses may not induce adequate anesthesia, and higher doses can produce hemodynamic changes, which can independently alter brain metabolism.

Since changes in intracerebral metabolites occur rapidly, whole brains were swiftly collected from the rats after anesthesia was completed and frozen in liquid nitrogen within 10 s. Frozen tissue was pulverized in a Cryo-Press™ using a stainless reservoir that had been chilled beforehand with liquid nitrogen. The level of metabolites such as lactate may increase at postmortem. However, since the time required for processing the specimen material was approximately the same between all the groups, we consider that this would not have greatly affected the data between the groups. Since the effects of anesthetic drugs differ depending upon the part of the brain being examined, we believe that it is important to evaluate the metabolite profile for each region. However, the experiments of this study were performed using whole brain samples. In future studies, changes in the metabolite profile for each brain region should be elucidated.

Glucose influenced the separation of the PCA score of the anesthetized group and the control group. It is well known that glucose is the energy source of the brain, and the carbon chain of glucose is incorporated via the tricarboxylic acid cycle into the neurotransmitters glutamate, aspartate, GABA, and glutamine [17]. On the micro level, the activity of nerve cells is made up of the transmission of neurotransmitters at the synapse. Brain activity occurs through the release of glutamate, an excitable amino acid, from the nerve endings. More specifically, when considering brain activity from the metabolism perspective, the metabolism of substances such as glutamate, aspartate, and GABA reflects brain metabolism, and ultimately, cerebral function. Anesthetic drugs cause changes in cerebral blood flow, cerebral metabolic rate (CMR), electroencephalogram in a dose-dependent manner [18], [19]. Propofol administration causes a decrease in both cerebral blood flow and CMR while isoflurane decreases CMR, but increases cerebral blood flow due to its cerebrovasodilating effect [19]–[21].

Apart from glucose, acetate, NAA, and choline were identified as the metabolites that contributed to the separation of PCA scores between the propofol and control groups. The metabolite choline is required for synthesis of the neurotransmitter acetylcholine, and phosphatidylcholine, a major constituent of membranes. NAA is a free amino acid present in the neuronal cells at a high concentration. Its function is poorly understood, but it is believed to act as an osmolyte, as a storage form of aspartate, and as a precursor of NAAG, as well as having a variety of other functions [22], [23]. It has been reported that the NAA level may reflect neuronal dysfunction. For example, NAA decreases gradually after the development of cerebral infarction [24] where it is broken down into aspartate and acetate by the enzyme aspartoacylase.

In our study, acetate was one of the primary factors involved in the finding that the PCA score of propofol was separate from the isoflurane and control groups. We believe that one of the reasons for the increase in intracerebral acetate levels was that propofol administration enhanced the *in vivo* production of acetate. Another reason may be that acetate is primarily metabolized by glial cells, and therefore, there might have been a lowered metabolic reaction as a result of anesthesia. The subsequent metabolism of acetate results in the production of adenosine, which has a number of effects in the central nervous system. In addition, it has been reported that acetate decreases the minimum alveolar concentration of inhalational anesthetics [25]. It is possible that acetate influences the interaction of inhalational anesthetics and intravenous anesthetic. The rise in intracerebral acetate levels resulted from propofol administration. However, the mechanism underlying this increase requires further elucidation.

Acetate, glutamate, glutamine and glycine were identified as factors separating the PCA scores of the propofol and isoflurane groups. Glutamate and glycine are known to play a role in the anesthetic mechanism. Glutamate acts as an excitatory neurotransmitter [26], while isoflurane reduces depolarization-evoked glutamate release in cortical brain slices in a dose-dependent manner [27]. Glycine is a simple amino acid that acts as an inhibitory neurotransmitter and is distributed in the central nervous system, and glycinergic inhibitory interneurons are known as a site of action of propofol [28], [29].

In our study, no separation of the PCA scores was observed at 2 h and 6 h when performing isoflurane anesthesia. Moreover, even during anesthesia for extended periods of time, there were only small variations in the metabolite profile of the brain when using this anesthetic. Isoflurane appears to be an anesthetic that minimally influences holistic brain metabolism. In the case of propofol, PCA scores became very separated from the controls over time. No significant differences in the hemodynamics between the propofol group and the isoflurane group were observed, and one reason for this finding may be that the dose used for 6 h of anesthesia was an overdose. Therefore, further studies are required to examine brain metabolism t when administrating a varying dosage.

In the present study, we successfully visualized changes in brain metabolic phenotypes over time under anesthesia. Hydrophilic metabolite profiling of the brain showed that anesthesia with propofol or isoflurane exerted different effects on rat holistic brain metabolism. Metabolomic investigation of the effects of anesthetics on brain endogenous metabolites provides useful information with regard to the selection of anesthetics for clinical use and for the development of new anesthetics. In the current study, only a single anesthetics concentration was investigated. Anesthetics influence glucose metabolism in the brain depending on the concentration, and therefore, further experiments on other concentrations are necessary. In addition, we only investigated changes in brain metabolism during anesthesia. Changes in genes and proteins have been reported after anesthesia [30]–[33]. However, the status of brain metabolites after anesthesia is not presently understood and changes in brain metabolism after anesthesia should be investigated further.

## Materials and Methods

### Animals and Preparation

The animal protocol was approved by the Animal Experimental Ethical Review Committee of Nippon Medical School. Adult male Wistar rats (280–320 g) were randomized to five groups, each group consisting of seven rats. Propofol and isoflurane were administered to two groups each, for 2 h or 6 h. The remaining group (control) received no anesthesia.

For all rats, a catheter was inserted into the tail vein without anesthesia, and lactated Ringer's (LR) solution was infused continuously at 1 ml/100 mg/h. During the experiment, each rat was placed into a plastic cage (45×32×23 cm) supplied with an air-oxygen mixture (fraction of inspired oxygen [FiO_2_]  = 0.33) and allowed to breathe spontaneously, with body temperature maintained at 37°C with a heat lamp. The propofol groups were infused with 1% propofol at 40 mg/kg/h in LR solution. The isoflurane groups were anesthetized in the cage by inhalation of 1.2% isoflurane. Each control rat was placed into the plastic cage. They were cannulated in the tail vein, infused with LR solution, and sacrificed 2 h later. All anesthetized groups were killed immediately after anesthesia. Whole brains were extirpated, immediately frozen in liquid nitrogen within 10 s for consideration of the influence of time on brain metabolites, and stored at −80°C until further processing for a week.

### Physiological Parameters

Physiological variables were measured in the three separate groups not taken for brain metabolomic evaluation (n = 7 each), (one isoflurane group, one propofol group and a control group [lack of anesthesia]) (Table 1). For all rats, a catheter was inserted into the tail vein without anesthesia and LR solution was infused continuously at 10 ml/kg/h. During the experiment, each rat was placed into a plastic cage (45×32×23 cm) supplied with an air-oxygen mixture (fraction of inspired oxygen [FiO_2_]  = 0.33) and allowed to breathe spontaneously, with body temperature maintained at 37°C with a heat lamp. The anesthetics were administered as described in the PCA analysis group. We cannulated the left femoral artery to draw blood samples for measuring arterial PO_2_, arterial PCO_2_, arterial blood pH, plasma glucose concentrations, heart rate, and arterial blood pressure. In the propofol and isoflurane groups, anesthesia was maintained for an additional 6 h. After surgery, the control group was placed in a rat tunnel and was allowed to recover from anesthesia. Blood samples were taken at 2 h. In addition, the propofol group and the isoflurane group had blood samples taken 6 h after the induction of anesthesia.

### Sample Preparation

Neutral extraction was performed according to Yoshioka et al. [34]. The method designed by Folch [35] was originally designed for the extraction of hydrophobic substances, but this method is also useful for extracting low molecular weight hydrophilic organic compounds, essentially without any *in vitro* modifications, i.e., oxidation hydrolysis and decomposition. Frozen tissue samples weighing approximately 1.0 g were ground and pulverized into a fine powder in liquid nitrogen using a frozen cell crusher ‘Cryo-Press™’ (Microtec, Chiba, Japan), and homogenized in 10 ml of a chloroform/methanol (2∶1) mixture. After removing the residues by filtration, 1 ml of distilled water was then added to the filtrates. After thoroughly mixing, they were left to stand for 24 h at 4°C to separate the hydrophilic phase layer from the hydrophobic phase. The hydrophilic phase was evaporated in an evacuated centrifuge overnight. Dried extracts were reconstituted in 0.6 ml of deuterium oxide (D_2_O) (ISOTEC Inc., USA) containing 0.25 mM sodium (3-trimethylsilyl) tetradeuteriopropionate-2,2,3,3-d4 (TMSP) (MSD Isotopes, Montreal, Canada) and then were pipetted into 5-mm NMR tubes (Wilmad-LabGlass, Buena, NJ, USA) for subsequent NMR measurements. D_2_O provided a deuterium field frequency lock for the NMR spectrometer, while TMSP provided an internal chemical shift reference (δ = 0.00). The pH of the sample solutions was 7.2 to 7.4.

### Acquisition of Proton Nuclear Magnetic Resonance Data

Solution state ^1^H NMR spectroscopy was performed at a proton resonance frequency of 300 MHz using an ECX NMR spectrometer interfaced with a TH5 probe (normal geometry, auto tunable type) equipped with an automatic 16-position sample changer and Delta™ NMR processing and control software (version 4.3.2, JEOL Ltd., Tokyo, Japan). Standard one-dimensional ^1^H NMR spectra were acquired automatically from 400 scans at a probe temperature of 23°C using the macro program in Delta systems for automatic measurement for metabolomics supplied by JEOL. Raw free induction decays were acquired with the use of a single pulse with a 2.0 s relaxation delay between pulses, and were suppressed by presaturation, using a conventional presaturation pulse sequence for water proton signal suppression based on homo-gated irradiation and DANTE pulse sequence (presaturation time  = 2 s; DANTE pulse  = 8 µs; DANTE interval  = 0.1 ms; DANTE loop  = 185; DANTE attenuator  = 24 dB). Other conditions were as follows: radio frequency pulse width  = 4.9 µs; acquisition time  = 1.470 s; repetition time  = 3.47 s; spectral width  = 5,580 Hz; data points  = 8192 points; number of steady states transients  = 4.

### Data Processing and Reduction

The resultant spectra were processed using Alice2™, version 5.5 (JEOL DATUM Ltd., Tokyo, Japan). FIDs were subjected to an exponential weighting function of 0.5 Hz, Fourier transformed from the time to frequency domain, and then phased manually, followed by linear baseline correction and referencing to the TMSP singlet at 0.00 ppm.

To simplify the ^1^H NMR spectra of the tissues by means of data compression, all spectra were integrated between 0.5 and 9.5 ppm using an integration macro written within the Alice2 for Metabolome™, version 1.0 (JEOL DATUM Ltd., Tokyo, Japan) software package, which integrated the spectrum into 215 segments (buckets) with 0.04 ppm integral regions. Regions containing resonances of residual water (4.6 ppm to 5.0 ppm) were excluded before integration. Close inspection of the important integral regions for pattern recognition identified some cases where one resonance straddled two or more integral buckets, and therefore, these integral regions were corrected manually combining or extending the regions to include one resonance in a single integral region. Other regions that largely consisted of noise were excluded to produce more significant multivariate mapping. Finally, all the spectral data were reduced into 177 buckets, and to account for the bulk mass differences between samples, each spectral region was normalized to the sum of all of the integrals of the buckets.

The assignment of metabolites in the^ 1^H NMR spectra was made by comparing the proton chemical shifts with literature values [36] and by comparison with spectra of authentic compounds via an in-house spectral database.

### Multivariate Analysis and Statistical Analysis

The calculated results obtained from all measured spectra were exported to a spreadsheet as a text file, which was then used as the input into the pattern recognition/multivariate statistics software package for non biased metabolic profiling. Datasets were imported into ADMEWORKS/ModelBuilder™ software, version 4.5 (Fujitsu Kyushu Communications Systems Ltd., Fukuoka, Japan) software package. All the data were mean-centered and a multicollinearity test and genetic-algorithm were used to identify the buckets that included the resonances from the key metabolites, which differed among the groups. Using the selected bucket data, PCA was performed to discern the presence of inherent similarities between spectral profiles. Data were visualized using the PC score and loading plots. PCA was used to examine trends and clustering in an unsupervised manner. In this analysis, the algorithm calculates the maximum amount of correlated variation in a data set and scores each spectrum according to this variation along PC1. This procedure is repeated for other components until the majority of the variations in the data set are described. Being an unsupervised method, spectra are grouped according to the highest amount of variance in the data set, regardless of the group of each sample. For each type of analysis, data were visualized by plotting PC scores. In the plots, each point represents an individual sample. The plots allow the recognition of clusters of samples with similar scores. Each score plot has a loading profile associated with it, which helps in identifying the spectral regions (metabolites) responsible for the sample clustering observed.

An ANOVA test was used to compare the physiological variables between the experimental and control groups. P<0.01 was considered to be significant. Data are given as the mean ± SD.
